# Inhibition of thyroid hormone signaling protects retinal pigment epithelium and photoreceptors from cell death in a mouse model of age-related macular degeneration

**DOI:** 10.1038/s41419-019-2216-7

**Published:** 2020-01-13

**Authors:** Hongwei Ma, Fan Yang, Xi-Qin Ding

**Affiliations:** Department of Cell Biology, University of Oklahoma Health Sciences Center, Oklahoma City, OK USA

**Keywords:** Diseases, Neurodegenerative diseases

## Abstract

Age-related macular degeneration (AMD) is the leading cause of blindness in the elderly. Dry AMD is characterized by a progressive macular degeneration of the retinal pigment epithelium (RPE) and photoreceptors, and the RPE oxidative damage/dystrophy is at the core of the disease. Recent population/patients-based studies have shown an association of high free serum thyroid hormone (TH) levels with increased risk of AMD. This work investigated the effects of TH signaling inhibition on RPE and photoreceptor damage/cell death in an oxidative stress-induced mouse model of AMD. TH signaling inhibition was achieved by anti-thyroid drug treatment and oxidative stress was induced by sodium iodate (NaIO_3_) administration. Mice treated with NaIO_3_ showed severe RPE and photoreceptor cell death/necroptosis, destruction, oxidative damage, retinal stress, and reduced retinal function. Treatment with anti-thyroid drug protected RPE and photoreceptors from damage/cell death induced by NaIO_3_, reduced oxidative damage of RPE and photoreceptors, and preserved retinal function. Gene expression analysis showed that the NaIO_3_-induced RPE/photoreceptor damage/cell death involves multiple mechanisms, including cellular oxidative stress responses, activation of necroptosis/apoptosis signaling, and inflammatory responses. Treatment with anti-thyroid drug abolished these cellular stress/death responses. The findings of this study demonstrate a role of TH signaling in RPE and photoreceptor cell death after oxidative stress challenge, and support a role of TH signaling in the pathogenesis of AMD.

## Introduction

Age-related macular degeneration (AMD) is the leading cause of blindness in elderly, exhibiting complex interplay of genetic and environmental factors^1,2^. There are two types of AMD, the dry and wet forms. Dry AMD, also known as geographic atrophy, is a form of slowly progressing geographic atrophy of the macula, and comprises a majority of AMD cases (∼90%), whereas wet AMD rapidly progresses to blindness and involves the abnormal formation of blood vessels in the macula. Dry AMD is characterized by a progressive macular degeneration of the retinal pigment epithelial (RPE) cells and photoreceptors, lipofuscin (A2E) accumulation, and drusen formation. It is generally recognized that multiple factors, including aging, oxidative stress, chronic inflammation, and genetic defects, are involved in the RPE and photoreceptor dystrophies/AMD lesions. However, oxidative stress/damage to the RPE has been recognized as the core pathogenic lesion of the disease^2–4^.

Thyroid hormone (TH) signaling regulates numerous physiological functions, including cell growth, differentiation, and metabolic homeostasis. In the eye, TH signaling regulates cone opsin expression^5,6^ and cone photoreceptor viability^7–10^. Recently, TH signaling has been implicated in the pathogenesis of AMD. The prospective population-based studies showed that higher free serum TH values were associated with increased risk of AMD^11–13^. The patient population-based study also showed a high association between thyroidopathy and AMD^14,15^. These findings suggest an association of TH signaling with AMD. Indeed TH signaling has been linked to other types of neurodegenerative conditions, including Alzheimer’s disease^16,17^. The present work investigated the effects of TH signaling inhibition on RPE and photoreceptor damage/cell death in a mouse model of AMD induced by sodium iodate (NaIO_3_). We found that treatment with anti-thyroid drug protected RPE and photoreceptors from oxidative damage and cell death/necroptosis induced by NaIO_3_ and preserved retinal function. Moreover, treatment with anti-thyroid drug abolished NaIO_3_-induced upregulation of the genes involved in cellular stress responses, inflammatory responses, and cell death signaling. The findings of this study demonstrate a role of TH signaling in RPE and photoreceptor cell death induced by oxidative stress challenge and support a role of TH signaling in the development and progression of AMD.

## Results

### Treatment with anti-thyroid drug protected RPE and photoreceptors from damage and cell death induced by NaIO_3_

**Fig. 1:**
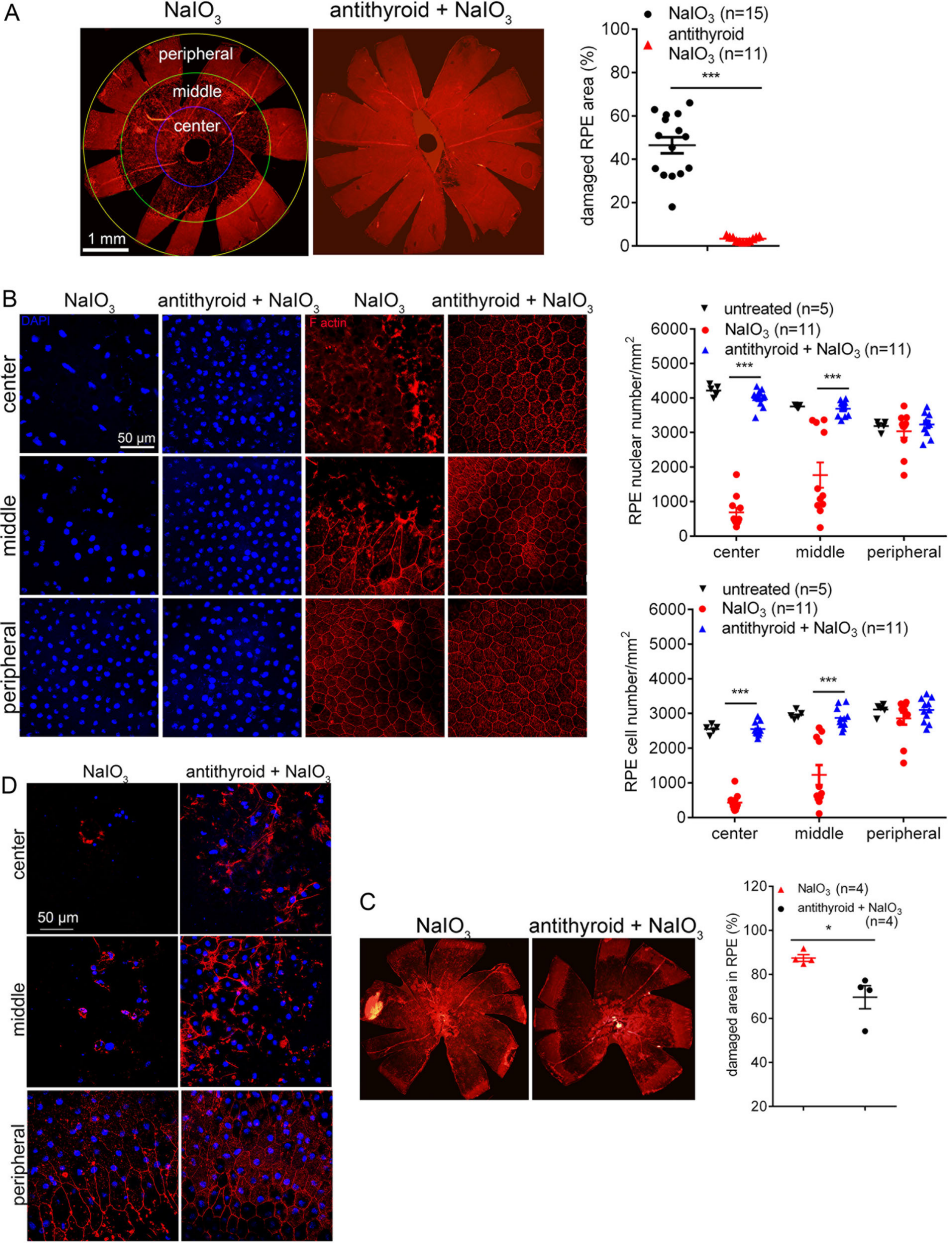
Treatment with anti-thyroid drug protected RPE from damage and cell loss induced by NaIO_3_. RPE morphology and cell loss were evaluated by phalloidin staining for F-actin and DAPI staining for nucleus on RPE whole mounts at 2–3 days post-NaIO_3_ injection. **a**, **b** Shown are RPE morphology evaluations in P30 mice. **a** Shown are representative low magnification images of phalloidin staining and corresponding quantitative analysis of the damaged area in the RPE. **b** Shown are representative high magnification images of phalloidin staining and DAPI labeling taken at different regions of the RPE, and corresponding quantitative analysis of RPE cell numbers and RPE nuclear numbers. Data represented the mean ± SEM for 5–15 mice per group (***p < 0.001). **c**, **d** Shown are RPE morphology evaluations in 17-month-old mice. **c** Shown are representative low magnification images of phalloidin staining and corresponding quantitative analysis of the damaged area in the RPE. **d** Shown are representative high magnification images of phalloidin staining and DAPI labeling taken at different regions of the RPE. Data represented the mean ± SEM for four mice per group (*p < 0.05).

A single treatment of NaIO_3_ induces RPE and photoreceptor oxidative damage/cell death, mimicking the feature and progression of AMD^18–20^. This model has been commonly used to study RPE and photoreceptor oxidative damage in AMD. To determine the role of TH signaling in oxidative stress-induced damage and cell death, we examined the effects of anti-thyroid treatment in mice that have been treated with NaIO_3_. C57BL/6J mice received anti-thyroid treatment via drinking water (1% sodium perchlorate monohydrate and 0.05% methomazole), beginning at postnatal day 20 (P20), received a single injection of NaIO_3_ (30 mg/kg, i.p.) at P30, and were then analyzed for RPE and photoreceptor damage/cell death at 3 days post-NaIO_3_ injection. ELISA analysis showed that the serum triiodothyronine (T3) level in anti-thyroid-treated mice were reduced by about 70%, compared with untreated controls (Supplementary Fig. 1). RPE morphology and cell loss were evaluated by phalloidin staining for F-actin and DAPI staining for nucleus on RPE whole mounts. As reported, treatment with NaIO_3_ induced severe damage of RPE, particularly in the central and middle regions (Fig. 1a). The RPE cells in NaIO_3_-treated mice were either completely lost in the severely damaged areas or were enlarged or irregularly shaped in the damaged and undamaged junction areas. Treatment with anti-thyroid drug profoundly preserved RPE cells from death. Evaluations of the low magnification images showed that the NaIO_3_ treatment caused damage in about 46% of the entire RPE area, and treatment with anti-thyroid drug nearly completely protected RPE cells (Fig. 1a). Evaluations of RPE cell numbers and nuclear numbers were performed on the high magnification images. The RPE cell numbers in the central and middle regions in NaIO_3_-treated mice were reduced by about 84% and 60%, respectively, compared with untreated controls, and anti-thyroid treatment completely prevented these reductions (Fig. 1b). Similar results were obtained from evaluations of the RPE nuclear numbers (Fig. 1b). The RPE layer integrity was also evaluated on eye cross sections with H&E staining. In NaIO_3_-treated mice, RPE layer in the middle and central regions exhibited thinner, gap and swelling, and possible macrophage infusion. Treatment with anti-thyroid drug greatly preserved RPE layer integrity, showing nearly normal RPE layer morphology (Supplementary Fig. 2A). Quantitative analysis revealed that the RPE nuclear numbers in NaIO_3_-treated mice was reduced by about 60%, compared with untreated controls, and the nuclear numbers in mice received anti-thyroid treatment was only reduced by about 10% (Supplementary Fig. 2B). Treatment with anti-thyroid drug alone did not induce any detectable changes in RPE morphology and cell loss (Supplementary Fig. 3).

In a separate experiment, we examined the effects of anti-thyroid drug treatment in aged mice. At 17 months of age, mice received anti-thyroid treatment, followed by a single injection of NaIO_3_ on the 10th day from the start of the anti-thyroid treatment, as described above. Mice were then analyzed for RPE morphology at 2 days post-NaIO_3_ injection. Evaluation of the low magnification images of phalloidin staining showed that the NaIO_3_ treatment caused damage in about 87% of the RPE area, and treatment with anti-thyroid drug reduced the damage to about 70% of the RPE area (Fig. 1c). Figure 1d shows high magnification images of phalloidin labeling and DAPI staining at different regions of RPE. Similar to young mice, treatment with anti-thyroid drug alone did not induce any detectable changes in RPE morphology and cell loss in aged mice (Supplementary Fig. 4).

**Fig. 2:**
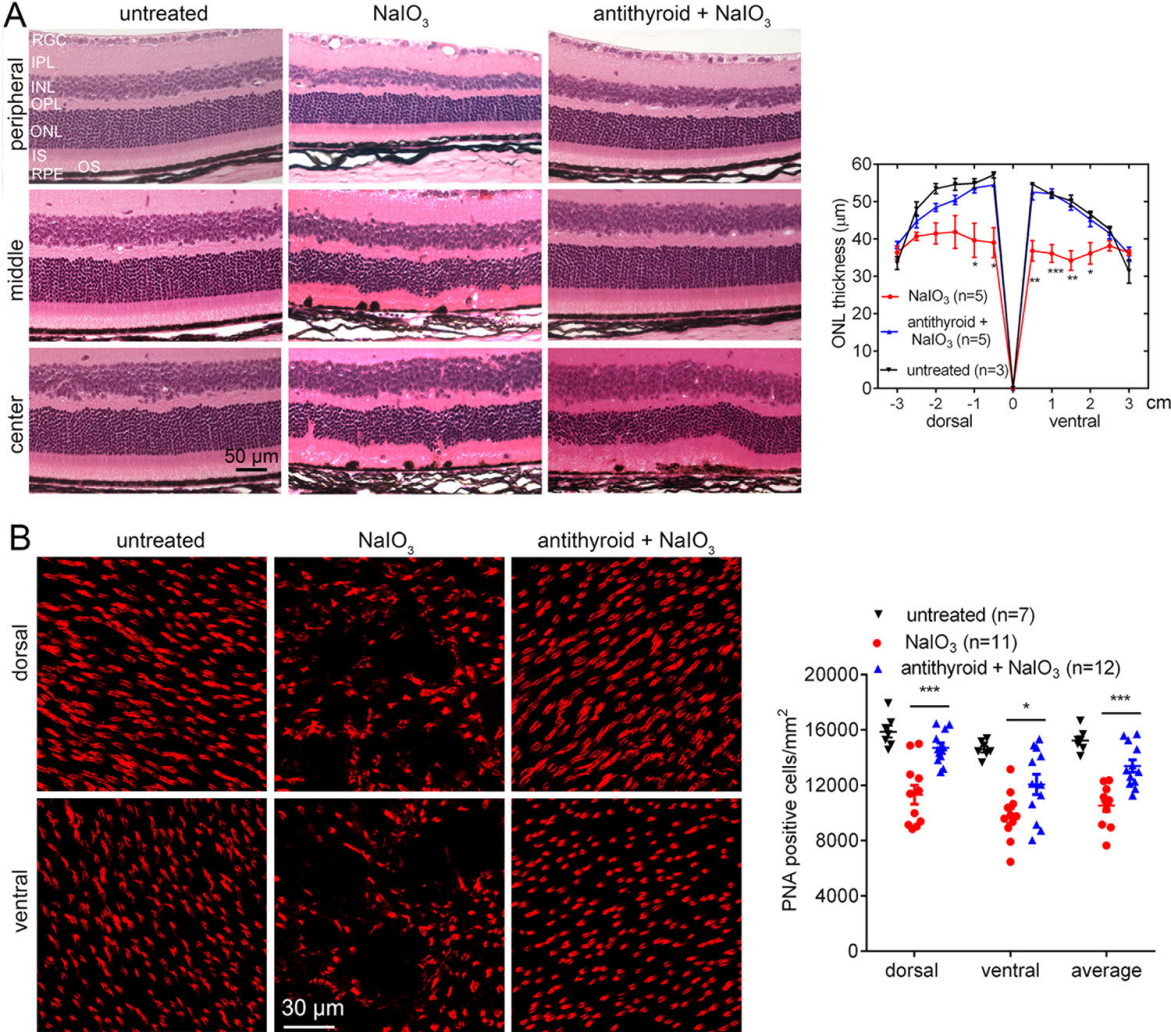
Treatment with anti-thyroid drug protected photoreceptors from cell loss/degeneration induced by NaIO_3_. Retinal morphology, photoreceptor layer integrity, and loss of photoreceptors were evaluated by light microscope and morphometric analysis at 3 days post-NaIO_3_ injection, and cone density was evaluated by PNA labeling on retinal whole mounts. **a** Shown are representative light microscopic images of H&E stained retinal sections, and corresponding quantitative analysis of ONL thickness in the dorsal and ventral regions. Data represented the mean ± SEM for 3–5 mice each group. **b** Shown are representative confocal images of PNA labeling on retinal whole mounts, and corresponding quantitative analysis. RPE, retinal pigment epithelial; OS, outer segment; IS, inner segment; ONL, outer nuclear layer; OPL, outer plexiform layer; INL, inner nuclear layer; IPL, inner plexiform layer; RGC, retinal ganglion cell. Data represented the mean ± SEM for 7–12 mice per group (*p < 0.05; **p < 0.01; ***p < 0.001).

Retinal morphology/photoreceptor numbers were evaluated on retinal cross sections and retinal whole mounts. The retinal section examinations showed severe damage of photoreceptor layer in mice after NaIO_3_ challenge. These mice displayed disorganized outer nuclear layer (ONL) and outer segment (OS) areas, reduced numbers/thickness of the ONL, and shortened OS (Fig. 2a). Treatment with anti-thyroid drug greatly preserved retinal morphology and prevented photoreceptor cell loss induced by NaIO_3_. The ONL thickness in the central retina was reduced by about 27% in mice after NaIO_3_ injection, and treatment with anti-thyroid drug nearly completely prevented the loss of photoreceptors (Fig. 2a). Peanut agglutinin (PNA) labeling on retinal whole mounts showed that NaIO_3_ injection reduced cone number by about 30%, compared with untreated controls, and treatment with anti-thyroid drug greatly preserved cones (Fig. 2b).

**Fig. 3:**
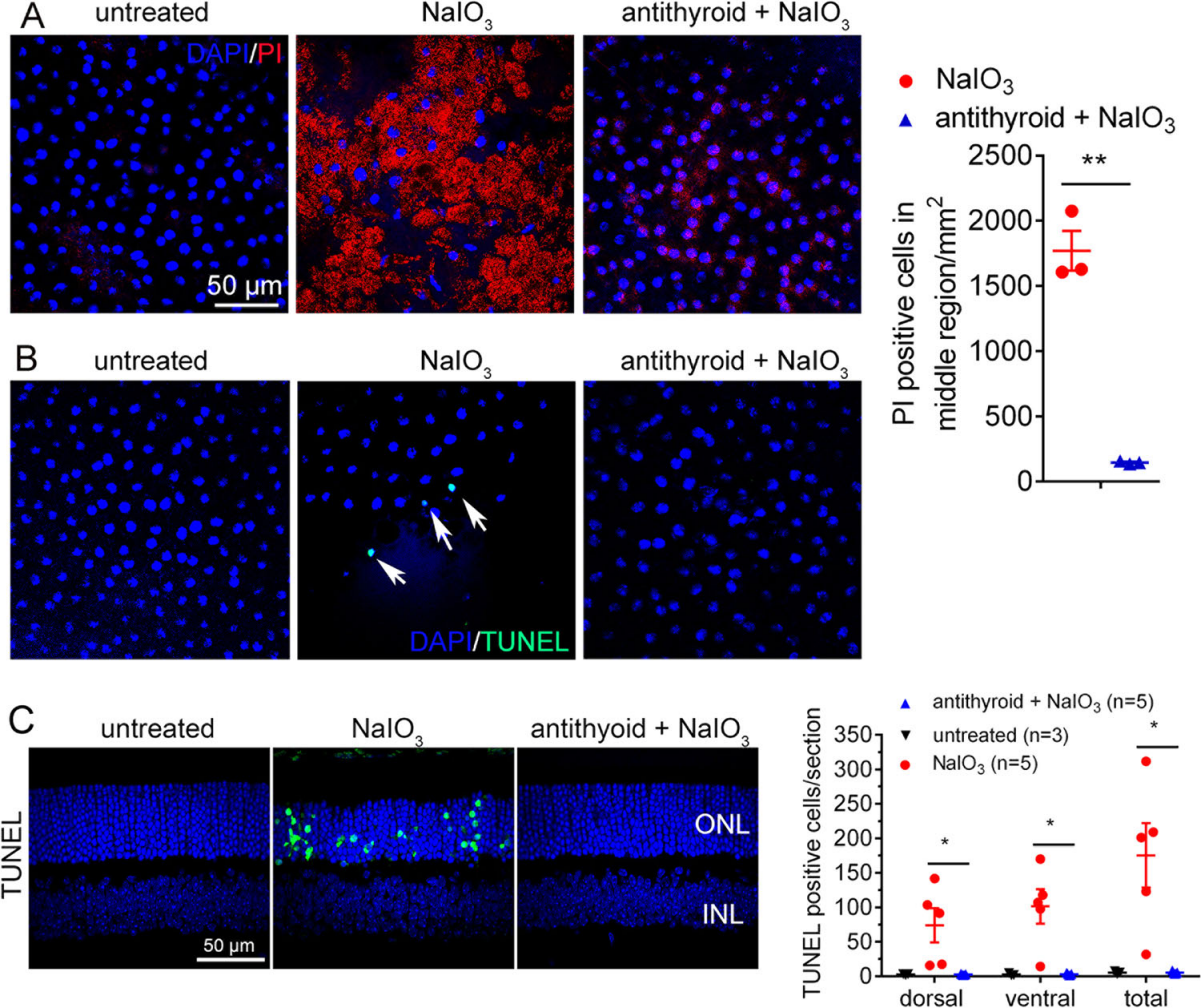
Treatment with anti-thyroid drug protected RPE cells and photoreceptor cells from necroptosis induced by NaIO_3_. **a**, **b** RPE cell necroptosis was evaluated by PI staining and TUNEL on the RPE whole mounts. Shown are representative images of PI staining at the middle region of the RPE at 2 days post-NaIO_3_ injection and correlating quantitative analysis (**a**), and representative images of TUNEL at the middle region of the RPE at 1 day post-NaIO_3_ injection (**b**). **c** Photoreceptor cell apoptosis was evaluated by TUNEL on the retinal sections at 3 days post-NaIO_3_ injection. Shown are representative images of TUNEL and correlating quantitative analysis. ONL, outer nuclear layer; INL, inner nuclear layer. Data represented the mean ± SEM for 3–5 mice per group (*p < 0.05, **p < 0.01).

The effects of anti-thyroid drug treatment were further evaluated by examining the molecular hallmarks of cell death. Propidium iodide (PI) staining has previously indicated NaIO_3_-induced RPE cell necroptosis, with abundant PI staining at 2 days post-NaIO_3_ injection^18^. At 2 days post-injection, we performed a retro-orbital PI injection for PI staining, and observed that NaIO_3_ treatment induced remarkable PI staining, while treatment with anti-thyroid drug nearly completely abolished the staining (Fig. 3a). Treatment with anti-thyroid drug alone did not induce any detectable PI staining (data not shown). Terminal deoxynucleotidyltransferase dUTP nick-end labeling (TUNEL)-positive RPE cells and TUNEL-positive photoreceptor cells have been detected at 1 to 3 days post-NaIO_3_ injection^18^. We performed TUNEL to evaluate the effects of anti-thyroid treatment. TUNEL-positive cells were detected on the RPE whole mounts at 1 day post-NaIO_3_ injection; anti-thyroid treatment abolished the TUNEL detection (Fig. 3b). Retinal section analysis revealed a large increase in the numbers of TUNEL-positive photoreceptor cells at 3 days post-NaIO_3_ injection; treatment with anti-thyroid drug eliminated the TUNEL labeling (Fig. 3c).

### Treatment with anti-thyroid drug protected RPE and photoreceptors from oxidative damage induced by NaIO_3_

**Fig. 4:**
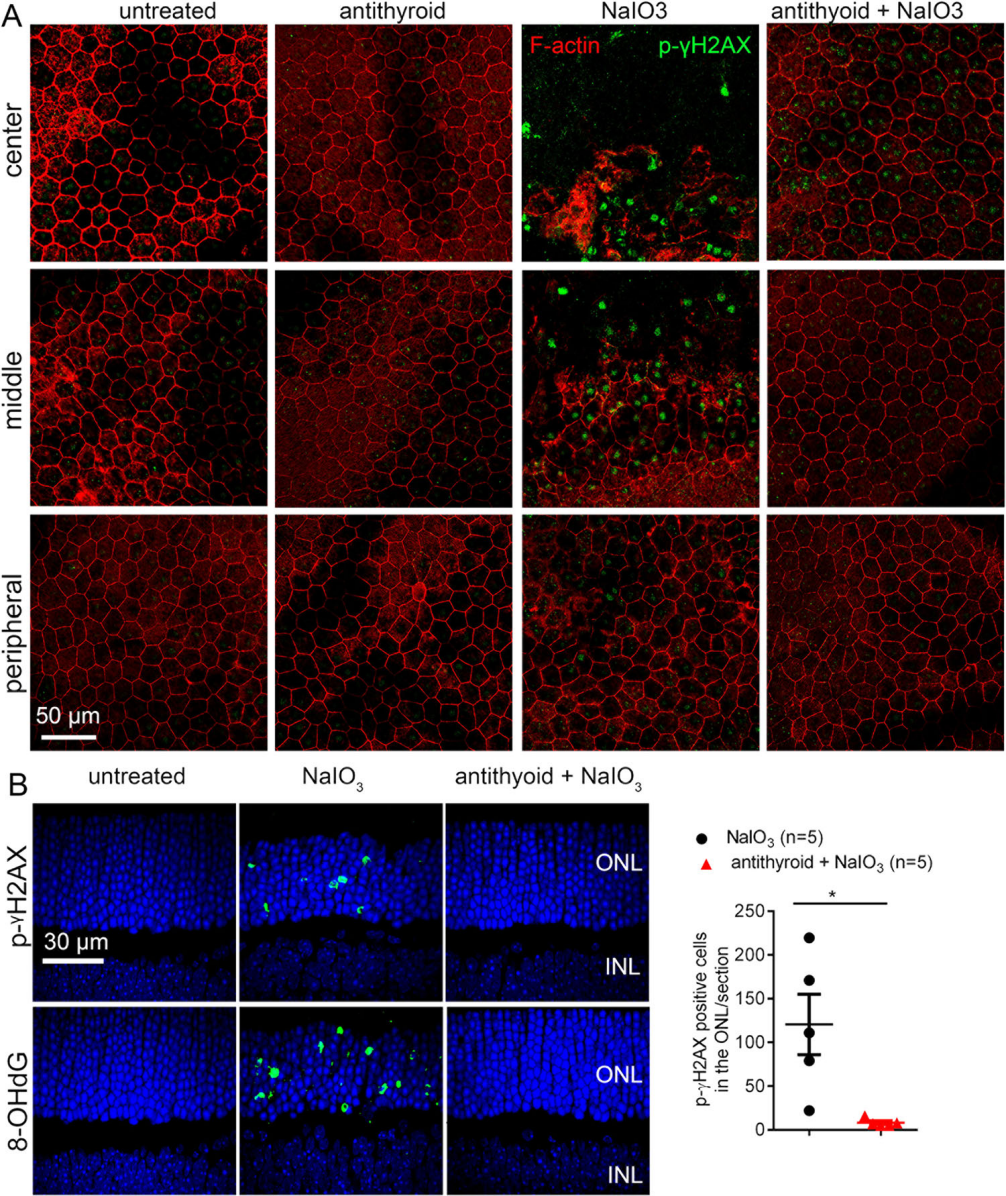
Treatment with anti-thyroid drug protected RPE and photoreceptors from oxidative damage induced by NaIO_3_. RPE and retinal oxidative damage were evaluated by immunofluorescence labeling of p-γH2AX and 8-OHdG on the RPE whole mounts and retinal sections at 3 days post-NaIO_3_ injection. **a** Shown are representative images of p-γH2AX immunofluorescence labeling on the RPE whole mounts. **b** Shown are representative images of p-γH2AX and 8-OHdG immunofluorescence labeling on the retinal sections, and corresponding quantitative analysis for p-γH2AX labeling. ONL, outer nuclear layer; INL, inner nuclear layer. Data represented the mean ± SEM for 5 mice per group (*p < 0.05).

We next examined the effects of anti-thyroid treatment on RPE and photoreceptor oxidative damage. Mice received anti-thyroid treatment and NaIO_3_ challenge, as described above, and were analyzed for RPE and photoreceptor oxidative damage at 3 days post-NaIO_3_ injection. RPE oxidative damage were assessed by immunofluorescence labeling of the DNA double strand break/damage markers p-γH2AX and 8-OHdG on the RPE whole mounts^21,22^. Mice that have been treated with NaIO_3_ showed significantly increased labeling of p-γH2AX, compared with untreated controls (Fig. 4a). The labeling signal was concentrated in the central and middle regions, correlating to the RPE damage pattern in which more cell death was found in these regions. Treatment with anti-thyroid drug greatly reduced NaIO_3_-induced elevation of p-γH2AX (Fig. 4a). Similar findings were obtained with p-γH2AX labeling on the retinal sections. Mice that have been treated with NaIO_3_ showed greatly increased labeling of p-γH2AX in the ONL layer, compared with untreated controls, and treatment with anti-thyroid drug completely abolished NaIO_3_-induced elevation of p-γH2AX (Fig. 4b). The effects of antithyroid drug on NaIO_3_-induced oxidative damage was also demonstrated by 8-OHdG labeling (Fig. 4b).

### Treatment with anti-thyroid drug suppressed Müller glia activation induced by NaIO_3_

**Fig. 5:**
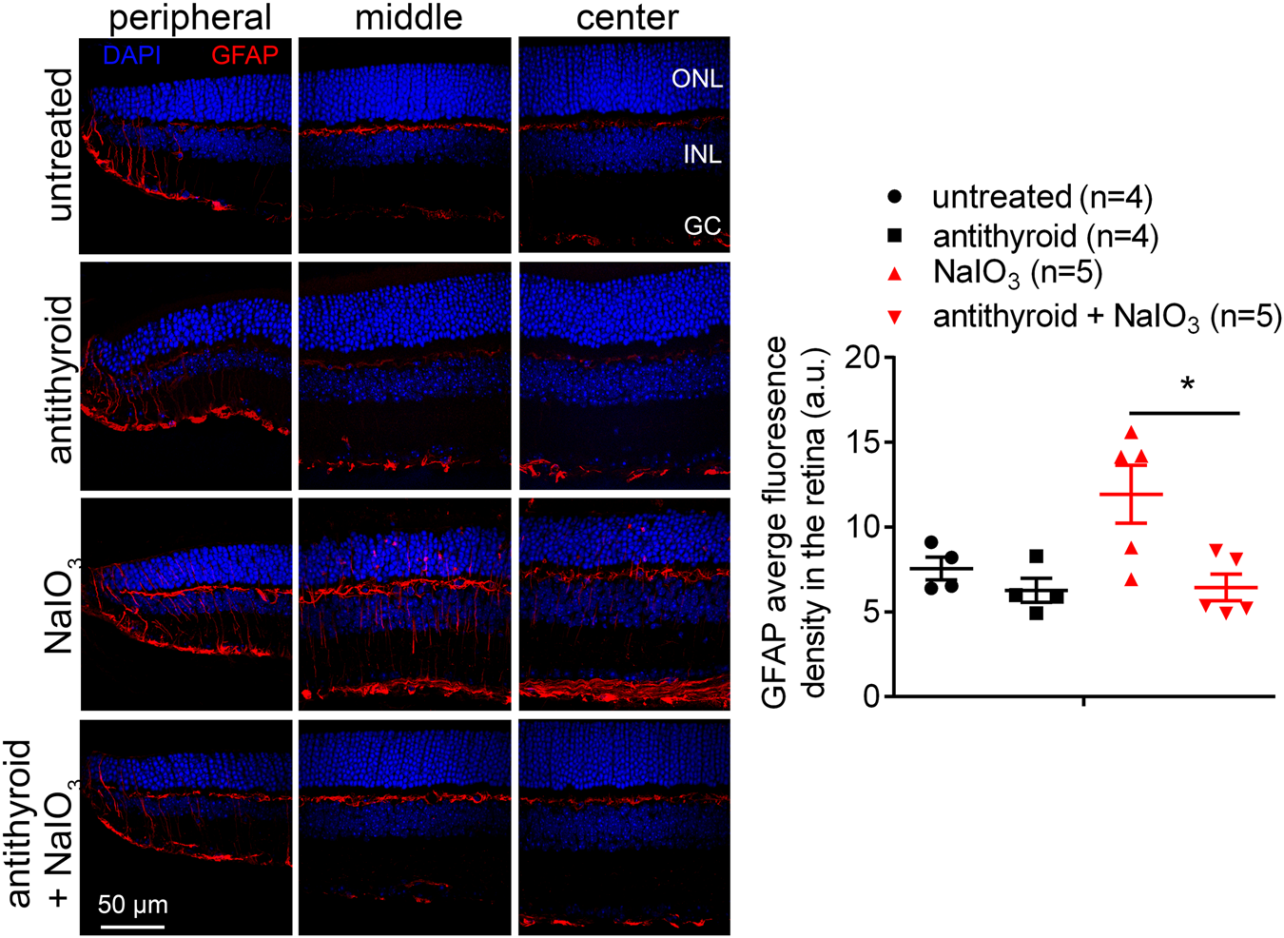
Treatment with anti-thyroid drug suppressed Müller glia activation induced by NaIO_3_. GFAP immunofluorescence labeling was performed on the retinal cross sections at 3 days post-NaIO_3_ injection. Shown are representative confocal images of immunofluorescence labeling of GFAP on the peripheral, middle, and central regions of the retinal sections and corresponding quantification of immunofluorescence intensity. ONL, outer nuclear layer; INL, inner nuclear layer; GC, retinal ganglion cell. Data represented the mean ± SEM for 4–5 mice per group (*p < 0.05).

Müller glia are known to activate in response to retinal stress by profound upregulation of glial fibrillary acidic protein (GFAP) in intermediate filaments. In this study, we examined the effects of anti-thyroid treatment on Müller glia activation. Mice received anti-thyroid treatment and NaIO_3_ challenge, as described above, and were analyzed for Müller glia activation at 3 days post-NaIO_3_ injection. Retinal cross sections were analyzed for expression of GFAP by immunofluorescence labeling. Quantitative analysis of the immunofluorescence intensity showed that the NaIO_3_ treatment increased expression of GFAP by about 67%, compared with untreated controls, and treatment with anti-thyroid drug completely abolished the NaIO_3_-induced GFAP expression (Fig. 5).

### Treatment with anti-thyroid drug preserved retinal function in mice treated with NaIO_3_

**Fig. 6:**
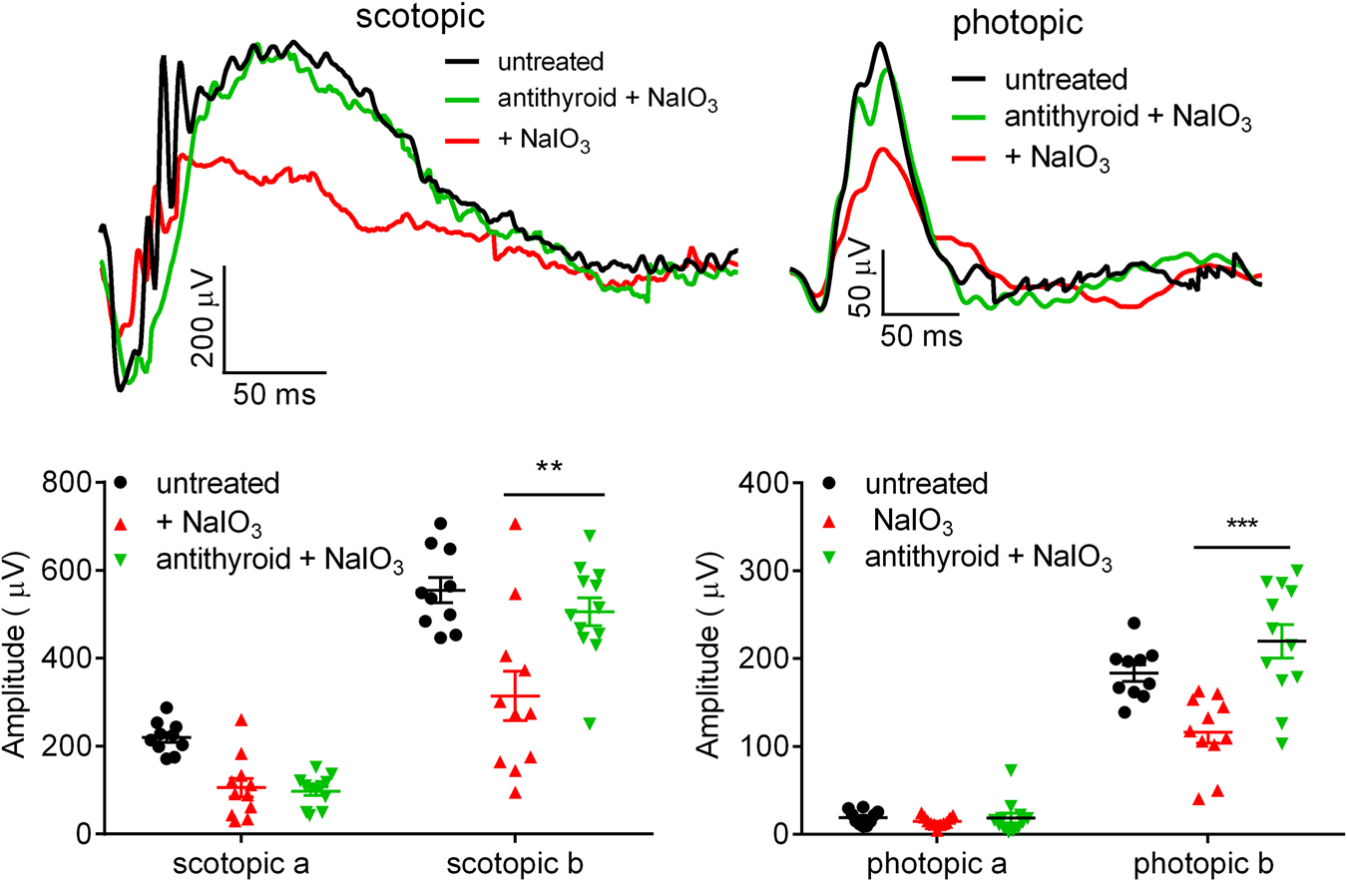
Treatment with anti-thyroid drug preserved retinal function in mice treated with NaIO_3_. Retinal light responses at 3 days post-NaIO_3_ injection were evaluated by ERG analysis. Shown are representative scotopic and photopic ERG recording waves and quantification of the ERG recordings. Data represented the mean ± *SEM* for 10–12 mice per group (**p < 0.01; ***p < 0.001).

We also examined the effects of anti-thyroid treatment on retinal function. Mice received anti-thyroid treatment and NaIO_3_ challenge, as described above, and were analyzed for retinal function by electroretinogram (ERG) recordings at 3 days post-NaIO_3_ injection. NaIO_3_ treatment reduced scotopic a- and b-wave amplitudes by about 55 and 44%, respectively, compared with untreated controls, and treatment with anti-thyroid drug significantly preserved scotopic b-wave but not a-wave responses (Fig. 6). Similarly, NaIO_3_ treatment reduced photopic b-wave amplitudes by about 39%, compared with untreated controls, and treatment with anti-thyroid drug completely preserved photopic b-wave responses (Fig. 6).

### Treatment with anti-thyroid drug reversed the NaIO_3_-induced gene expression upregulation in the RPE and retina

**Fig. 7:**
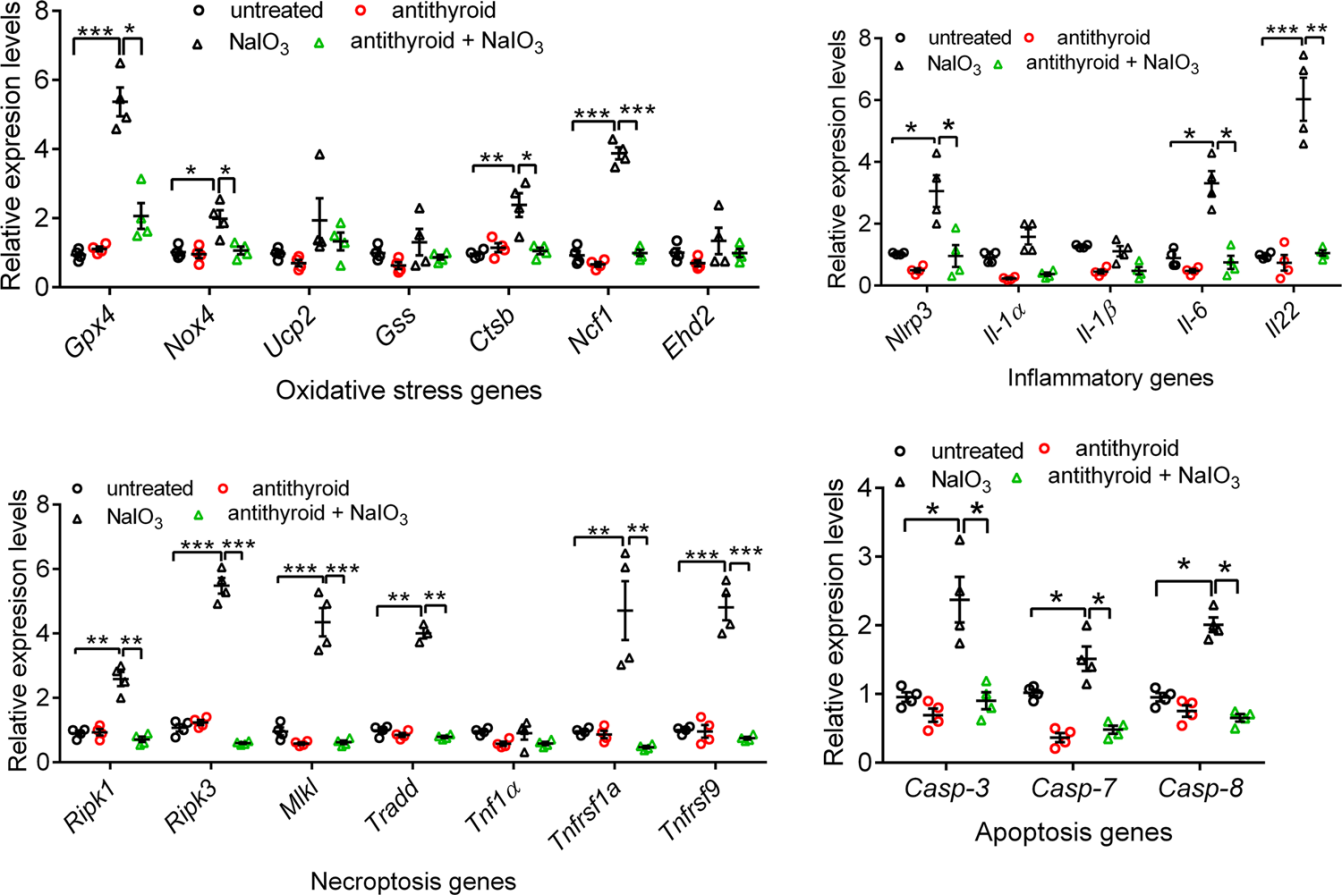
Treatment with anti-thyroid drug reversed the NaIO_3_-induced gene expression upregulation in the RPE. Expression levels of the genes involved in cellular stress responses and death signaling were examined in the RPE by qRT-PCR at 1 days post-NaIO_3_ injection. Shown are expression levels of the genes involved in oxidative stress responses, apoptosis/necroptosis pathways, and inflammatory responses. Data represented the mean ± SEM for 4 assays using RPE prepared from 5–7 mice per group (*p < 0.05, **p < 0.01, and ***p < 0.001).

**Fig. 8:**
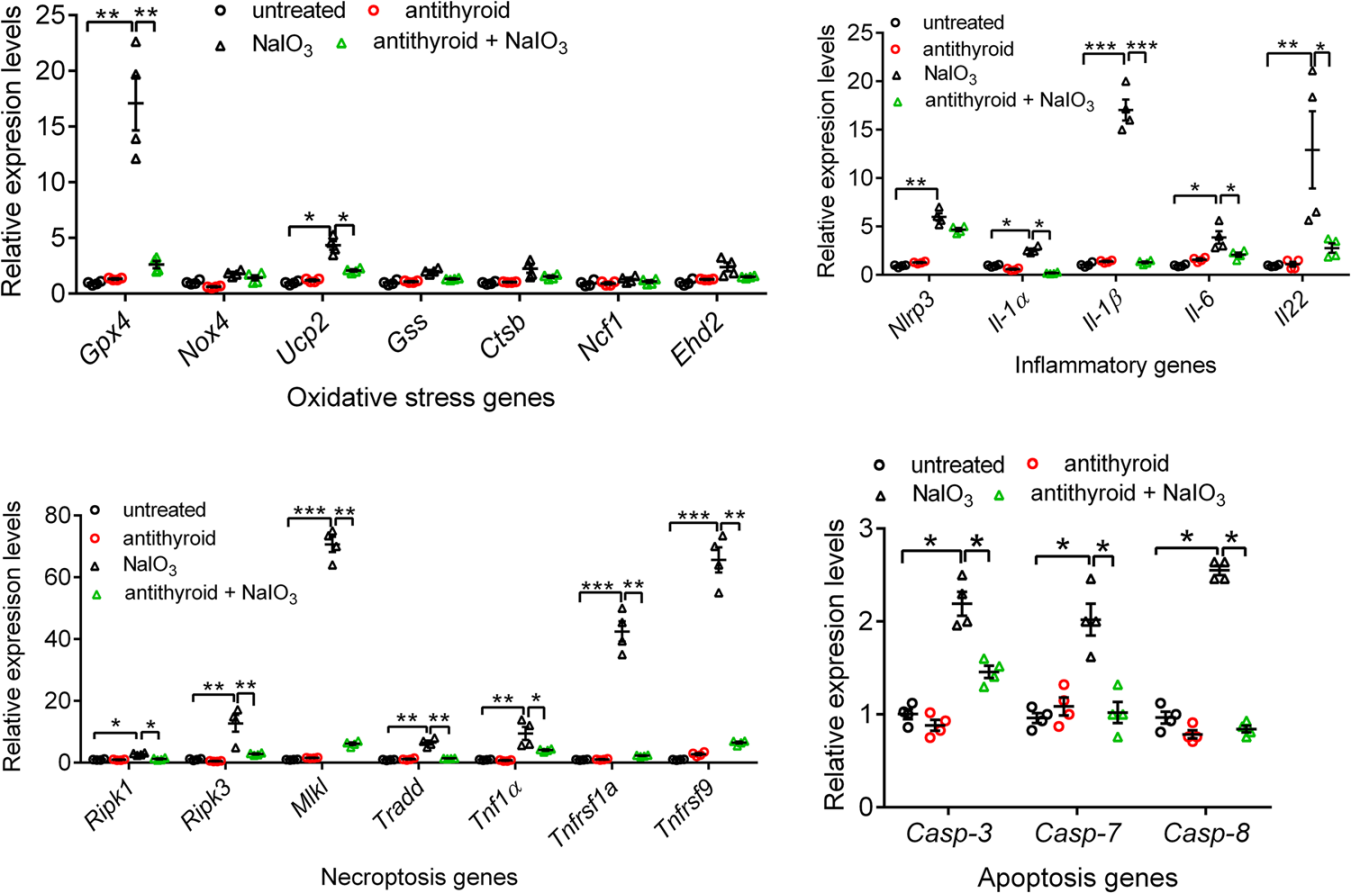
Treatment with anti-thyroid drug reversed the NaIO_3_-induced gene expression upregulation in the retina. Expression levels of the genes involved in cellular stress responses and death signaling were examined in the retina by qRT-PCR at 1 days post-NaIO_3_ injection. Shown are expression levels of the genes involved in oxidative stress responses, apoptosis/necroptosis pathways, and inflammatory responses. Data represented the mean ± SEM for 4 assays using retinas prepared from 3 mice per group (*p < 0.05, **p < 0.01, and ***p < 0.001).

To explore the mechanisms underlying TH signaling suppression-induced protection, we examined expression of the genes involved in oxidative stress responses, including *Gpx4*, *Nox4*, and *Ncf1*, apoptosis/necroptosis pathways, including *Casp3*, *Casp7*, *Casp8*, *Tnfrsf1α*, *Ripk1*, *Ripk3*, *Mik1*, and inflammatory responses, including *Nirp3*, *Il-1α/β*, *Il-6*, and *Il22*. Mice received anti-thyroid treatment and NaIO_3_ challenge, as described above, and were analyzed for gene expression in the RPE and retina by qRT-PCR at 1 day post-NaIO_3_ injection. NaIO_3_ treatment significantly induced expression of these genes in the RPE (Fig. 7) and retina (Fig. 8), and treatment with anti-thyroid drug nearly completely abolished the upregulation of the gene expression induced by NaIO_3_ (Figs. 7–8). Treatment with anti-thyroid drug alone did not induce significant expression alteration of these genes (Figs. 7–8). In a comparison between RPE and retinas, we found that the apoptotic genes were similarly upregulated in the RPE and retina, there were more oxidative stress response genes upregulated in the RPE than that in the retina, and there was more significant elevation of the necroptosis genes and inflammatory genes in the retina than that in the RPE.

## Discussion

NaIO_3_ induces RPE dystrophies/photoreceptor degeneration, primarily by inducing oxidative stress, mimicking the feature and progression of AMD^23^. A single administration of NaIO_3_ (by i.v., i.p. or intraocular injection) selectively induces RPE oxidative damage in experimental animals in a concentration-dependent manner, with more severe damage in the central and middle regions of the RPE^24,25^. Photoreceptors degenerate as results of RPE cell loss and the drug’s direct action. NaIO_3_ challenge has been used in various animal models, including mouse^26–28^, rat^29,30^, rabbit^30,31^, sheep^32^, cat^33^, and swine^30,34,35^, to study RPE/photoreceptor oxidative damage in AMD, and evaluate the novel therapeutic interventions. In the present study, we used this model to investigate the effects of TH signaling inhibition. As expected, a single injection of NaIO_3_ caused severe damage of RPE and photoreceptors, activated multiple cellular stress/death pathways, and impaired retinal function. Treatment with anti-thyroid drug nearly completely preserved RPE and photoreceptors from damage/cell death, reversed gene expression alterations, and partially preserved retinal function. These findings demonstrate a role of TH signaling in RPE and photoreceptor cell damage/death induced by NaIO_3_/oxidative stress. The observed effects from anti-thyroid treatment likely resulted from the reduced TH levels in the circulation (anti-thyroid drug treatment reduced the serum T3 level by about 67%; see Supplemental Fig. 1) and the subsequently reduced TH signaling in the target tissues/cells. Investigation using mice with deficiency of TH receptors will provide further insight.

We also examined the effects of anti-thyroid drug treatment in aged 17-month-old mice. We found that the aged mice displayed much more severe RPE damage/loss after NaIO_3_ challenge (87% damage area, Fig. 1c) than did the young mice (46% damage area, Fig. 1a). This finding suggests that the aged mice were more sensitive to NaIO_3_/oxidative challenge. In addition, the protective effects from anti-thyroid treatment were less significant in the aged mice than in the young mice. Anti-thyroid treatment nearly completely protected RPE from damage/cell loss in the young mice (see Fig. 1a), but only partly, though significantly, protected RPE cells in the aged mice. The difference in the protection efficiency might be associated with much more severe damage and/or less responsiveness to the anti-thyroid treatment in the aged mice.

Because photoreceptor damage/cell death after NaIO_3_ administration is a consequence of the loss/dysfunction of RPE and the direct harmful action of NaIO_3_^36,37^, the observed photoreceptor protection was likely achieved *via* the indirect protection from reduced RPE damage and the direct protection. TH regulation of cone survival has been well documented previously. Excessive TH signaling causes cone degeneration, whereas suppression of TH signaling protects cones in mouse models of inherited retinal degeneration^7–10,38^. Compared with the understanding of TH regulation of cone survival, we know little about TH regulation of rod survival. This work for the first time shows rod protection by TH signaling suppression in a mouse model of retinal degeneration, demonstrating a regulation of TH signaling in rod viability, which merits further investigation. The present study also demonstrates a protection of retinal function by TH signaling suppression. Anti-thyroid treatment completely reversed the reduction of ERG b-wave amplitudes induced by NaIO_3_. This functional rescue was likely resulted from the protection of retinal morphology/reduced photoreceptor cell death. However, the anti-thyroid treatment did not rescue the scotopic a-wave, which reflects the responses of rods, though a near complete rescue of retinal morphology and rod number/ONL thickness was achieved (see Fig. 2). The reason behind this observation is unclear at this time. It may suggest a critical regulatory role of TH signaling in the rod function and needs further investigation.

Although oxidative stress/damage serves as an initiating factor, NaIO_3_-induced RPE dystrophies/photoreceptor death is a multifactorial condition, triggered by oxidative stress, associated with inflammatory responses, and involves both caspase-dependent and caspase-independent (including necroptosis) mechanisms^18–20,30,36,39,40^. Inhibitors of caspases and necroptotic signaling pathways inhibit RPE and photoreceptor cell death induced by NaIO_3_ in vivo and in vitro^18–20,30,39^. Consistent with previous reports, this study shows that a single injection of NaIO_3_ induced upregulation of the genes involved in oxidative stress responses, inflammatory responses, and cellular necroptotic/apoptotic signaling. The local inflammatory responses/innate immune responses were also shown by activation of Müller glia/up-regulation of GFAP. There are a few interesting findings in the gene expression alterations: the apoptotic genes were similarly upregulated in the RPE and retinas; more oxidative stress response genes were upregulated in the RPE, relative to that in the retina; and more significant elevation of the necroptosis genes and inflammatory genes was observed in the retina, relative to that in the RPE. These observations support the view that the oxidative stress responses are the predominant reactions in the RPE whereas the necroptosis/inflammatory responses are the predominant reactions in the retinas. Nevertheless, treatment with anti-thyroid drug effectively suppressed expression of these genes in both RPE and retinas and abolished Müller cell activation. Thus, TH signaling inhibition-induced RPE and photoreceptor protection was likely achieved via multiple mechanisms, including suppression of oxidative stress responses, cell death signaling activity, and inflammatory responses. The question of how inhibition of TH signaling leads to suppression of these different cellular stress responses/death activities remains to be addressed. Because TH signaling plays a pivotal role in mitochondrial metabolism/homeostasis and reactive oxygen species production, one would expect that the anti-oxidative stress effects/protection of mitochondrial homeostasis might be at the core of the TH signaling inhibition-induced protection. Investigations on the TH regulation of RPE and photoreceptors with a focus on the mitochondrial homeostasis/stress might be particularly significant.

AMD is a multifactorial disorder, involving apoptosis/necroptosis of both RPE and photoreceptors, triggered by oxidative stress and worsened by inflammatory responses. This work shows that TH signaling inhibition protected RPE and photoreceptor cells from oxidative damage/cell death in an oxidative stress mouse model of AMD, accompanied by suppression of the upregulation of the genes involved in oxidative stress and inflammatory responses. Results from this animal model study are in line with the clinical findings showing a correlation of high free serum TH levels with increased risk of AMD, and support a role of TH signaling in the pathogenesis of AMD. Because both RPE and photoreceptors are involved in the disease pathogenesis and are protected by anti-thyroid treatment, inhibition of TH signaling may provide dual benefits in the management of AMD.

In summary, this work investigated the effects of TH signaling inhibition on RPE/photoreceptor cell death and retinal function in an NaIO_3_-induced mouse model of AMD. We show that the anti-thyroid treatment reduced RPE/photoreceptor oxidative damage/cell death, protected retinal function, and suppressed upregulation of the genes involved in cellular oxidative stress responses, cell death pathways, and inflammatory responses. The results of this study demonstrate a role of TH signaling in the RPE/photoreceptor cell death induced by oxidative stress challenge, and support a role of TH signaling in the pathogenesis of AMD. Further investigation on the regulation of TH signaling in the RPE and photoreceptor survival will help understand how suppression of TH signaling leads to protection and whether targeting TH signaling has therapeutic significance for AMD.

## Materials and methods

### Mice and reagents

C57BL/6J mice were obtained from the Jackson Laboratory and used in this study. Mice were maintained under cyclic light (12-h light–dark) conditions. Cage illumination was 7-foot-candle during the light cycle. All animal maintenance and experiments were approved by the local Institutional Animal Care and Use Committee (University of Oklahoma Health Sciences Center) and conformed to the guidelines on the care and use of animals adopted by the Society for Neuroscience and the Association for Research in Vision and Ophthalmology. Mice of either sex were used in the experiments. Mice were randomly assigned, within a litter, for the drug treatment or vehicle/untreated experiments; littermate controls were used whenever possible; and no animals were excluded from the analysis. No blinding was carried out for animal experiments.

Alexa Fluor^®^ 594 phalloidin (Catalog#: A12381) and Alexa Fluor^®^ 488 donkey anti-rabbit IgG (Catalog#: A21206) were purchased from Life Technologies; DAPI (4,6-Diamidino-2-phenylindole, Catalog#: D9542), NaIO_3_ (Catalog#: S4007), and PI (Catalog#: 537059) were purchased from Millipore Sigma; biotinylated PNA (Catalog#: B-1075) was purchased from Vector Labs; GFAP antibody (Catalog#: Z0334) was purchased from DAKO; p-γH2AX antibody (Catalog#: NB100–2280) was purchased from Novus Biologicals; and 8-OHdG (E8) antibody (Catalog#: sc393871) was purchased from Santa Cruz Biotechnology, Inc.

### Anti-thyroid treatment and NaIO_3_ injection

Anti-thyroid treatment and NaIO_3_ injection were performed as described previously^8,37^. Briefly, mice received anti-thyroid treatment *via* drinking water (1% sodium perchlorate monohydrate and 0.05% methomazole), beginning at P20, and received a single injection of NaIO_3_ (30 mg/kg, i.p.) at P30. These mice were then analyzed for RPE and photoreceptor damage/cell death and retinal function at 3 days post-NaIO_3_ injection, and for gene expression alterations at 1 day post-NaIO_3_ injection.

### Measurement of T3 in circulation

Serum T3 levels were analyzed using a mouse/rat T3 ELISA kit (Catalog#: T3043T-100, Calbiotech) with a total T3 detection limit at 0.25 ng/mL, as described previously^8^. Briefly, 25 μL of serum samples and standards with different T3 concentrations were added into the assigned wells, the assays were performed by following the manufacturer’s instruction, and the absorbance of each well was read at 450 nm (SpectraMax 190 Microplate Spectrophotometer, Molecular Devices). The standard curve was generated by using a three-parameter exponential nonlinear regression in Sigma-Plot software, and the sample T3 concentration was then calculated according to the three-parameter exponential equation.

### Eye preparation, immunofluorescence labeling, confocal microscopy, and retinal morphometric analysis

The RPE whole mounts were prepared for immunofluorescence labeling. Briefly, eyes were enucleated and fixed in 4% paraformaldehyde (PFA; Polysciences, Inc.) for 1 h at room temperature, followed by removal of the cornea, lens, muscles, and retina. The RPE sheets (the sclera-choroid-RPE sheets) were then fixed in 4% PFA for another 1 hour at room temperature, followed by wash (PBS, 5 min 3x) and blocking with 10% FBS in 0.5% Triton X-100 in PBS for 1 hour at room temperature. The RPE sheets were then stained with Alexa Fluor^®^ 594 phalloidin (1:40) for 30–45 min at room temperature and DAPI (1 ng/mL) for another 30 min at room temperature, followed by wash (PBS, 5 min 2x). The RPE whole mounts were made by transferring the sheets onto the slides, followed by mounting with Hard medium (H-1500, Vector Laboratories).

The retinal whole mounts and cross sections were prepared for immunofluorescence labeling, as described previously^8^. For retinal whole mount preparations, eyes were enucleated, marked at the superior pole with a green dye, and fixed in 4% PFA for 30 min at room temperature, followed by removal of the cornea and lens. The eyes were then fixed in 4% PFA in PBS for 4–6 h at room temperature, and retinas were isolated and the superior portion was marked for orientation with a small cut. For retinal cross sections, eyes were enucleated (the superior portion of the cornea was marked with green dye prior to enucleation) and fixed in Prefer (Anatech Ltd.) for 25–30 min at room temperature. Paraffin sections (5-µm thickness) passing vertically through the retina (along the vertical meridian passing through the optic nerve head) were prepared using a Leica microtome (Leica Biosystems). Immunofluorescence labeling was performed as described previously^8^. Briefly, retinal whole mounts or sections were blocked with Hanks’ balanced salt solution containing 5% BSA and 0.5% Triton X-100 for 1 h at room temperature or overnight at 4 °C. Prior to blocking, antigen retrieval was performed in 10 mM sodium citrate buffer (pH 6.0) for 30 min in a 70 °C water bath. Primary antibody incubation (p-γH2AX, 1:200; GFAP, 1:500; 8-OHdG, 1:200) was performed at room temperature for 2 h, followed by incubation with AlexaFluor-488 or −568, or FITC-conjugated secondary antibody. PNA immunohistochemistry was performed using biotinylated PNA (1:250) and streptavidin-Cy3 (1:500).

Low magnification images were taken under the Olympus MVX10 dissection microscope equipped with Image-Pro 6.3 software (Media Cybernetics, Inc.) and high magnification images were taken with an 60X objective on the FV1000 confocal laser scanning microscope equipped with FluoView imaging software (Olympus, Melville). ImageJ software (https://imagej.net/) was used to analyze the damaged area on the RPE whole mounts. For quantification of RPE cell numbers and RPE nuclear numbers, images from four quadrants in the central, middle and peripheral regions were counted and normalized to the number in one square millimeter. Evaluation of cone density on retinal whole mounts was performed as described previously^8,41^. For retinal morphometric analysis, retinal cross sections stained with hematoxylin and eosin (H&E) were used for morphometric analysis to evaluate ONL integrity/rod survival, as described previously^8,42^.

### PI staining and TUNEL assays

PI staining of RPE whole mounts was performed as described previously^18^. PI (0.5 μg) in 50 μl PBS was delivered through retro-orbital injection at 15 min before sacrificing the mice. The enucleated eye globes were fixed in 4% PFA for 1 hour, anterior part was removed, and the sclera-RPE was fixed in 4% PFA for one additional hour, followed by DAPI staining and fluorescence microscopy.

Terminal deoxynucleotidyltransferase dUTP nick-end labeling (TUNEL) was performed on paraffin-embedded retinal sections, using an in situ cell death fluorescein detection kit (Sigma-Aldrich, Catalog#: 11684795910), as described previously^43^. Immunofluorescence labeling was imaged using an Olympus FV1000 confocal laser-scanning microscope, and TUNEL-positive cells in the outer nuclear layer passing through the optic nerve were counted and averaged from at least 3 sections per eye from 3–5 mice per condition. TUNEL was also performed on RPE whole mounts. Briefly, eyes were fixed in 4% PFA for 1 hour. The sclera-RPE were then dissected and fixed in 4% PFA for one additional hour. After antigen retrieval performed in 10 mM sodium citrate buffer (pH 6.0) for 30 min at 70 °C, the sclera-RPE were permeabilized in 1% Triton X-100 in 10% FBS for 2 h at room temperature, followed by labeling using the *in situ* cell death fluorescein detection kit.

### Scotopic and photopic ERG recordings

Full-field ERG testing was carried out as described previously^44^. Briefly, after overnight dark adaptation, animals were anesthetized by intraperitoneal injection of 85 mg/kg ketamine and 14 mg/kg xylazine. ERGs were recorded using an LKC system (Gaithersburg, MD). Potentials were recorded using a platinum wire contacting the corneal surface through a layer of 2.5% methylcellulose. For assessment of scotopic responses, a stimulus intensity of 1.89 log cd s m^−2^ was presented to dark-adapted dilated mouse eyes in a Ganzfeld (GS-2000; Nicolet Instruments, Inc., Madison, WI). To evaluate photopic responses, mice were adapted to a 1.46 log cd s m^−2^ light for 5 min, then a light intensity of 1.89 log cd s m^−2^ was administered. Responses were differentially amplified, averaged, and stored using a Nicolet Compact-4® signal averaging system.

### RNA isolation and quantitative real-time PCR

The mouse RPE cells were isolated as described^45^. Total RNA preparation and reverse transcription were performed as described previously^46^. The gene encoding the mouse hypoxanthine guanine phosphoribosyl transferase 1 (*Hprt1*) was included as an internal control. Supplemental Table 1 shows the primers used. The quantitative real-time PCR (qRT-PCR) assays were performed using a real-time PCR detection system (iCycler; Bio-Rad Laboratories, Hercules, CA, USA), and the relative gene expression value was calculated based on the ΔΔCt method, as described previously^46^.

### Statistics

Results are expressed as means ± SEM of number of mice. Power analysis was performed to choose the sample size. The analysis indicates that a sample size of 3–6 mice/group for evaluations of retinal degeneration in the mouse retinas will provide at least 80% power (1-β) for a two-sided, two-sample *t*-test at a 0.05 alpha level. One-way ANOVA was used to analyze for significance within sets of data, and two-tailed Student’s *t*-test was used for differences between two groups of data. Differences were considered statistically significant when *P* < 0.05. Statistical tests for every figure are justified as appropriate. Data were analyzed and graphed using GraphPad Prism® software (GraphPad Software, San Diego, CA).

## Supplementary information

Supplementary Information

Supplementary Table 1

Supplementary Figure 1

Supplementary Figure 2

Supplementary Figure 3

Supplementary Figure 4
