# Supplementary material for: Inhibition of thyroid hormone signaling protects retinal pigment epithelium and photoreceptors from cell death in a mouse model of age-related macular degeneration
Source: Cell Death Dis. 2020 Jan 13;11(1):24. doi: 10.1038/s41419-019-2216-7 (PMC6957507; doi:10.1038/s41419-019-2216-7)
Supplement: Supplementary file 1 — Supplementary Information [file 41419_2019_2216_MOESM1_ESM.docx]

*For consideration for publication in Cell Death & Disease*

**Inhibition of Thyroid Hormone Signaling Protects Retinal Pigment Epithelium and Photoreceptors from Cell Death in A Mouse Model of Age-Related Macular Degeneration**

Hongwei Ma, Fan Yang, and Xi-Qin Ding*

Department of Cell Biology, University of Oklahoma Health Sciences Center, Oklahoma City, Oklahoma, USA

*To whom correspondence should be addressed: Xi-Qin Ding, Department of Cell Biology, University of Oklahoma Health Sciences Center, 940 Stanton L. Young Blvd., BMSB 553, Oklahoma City, Oklahoma 73104, USA; Phone: 1 (405) 271-8001 ext. 47966; Fax: 1 (405) 271-3548; E-mail: xi-qin-ding@ouhsc.edu

**Supplementary Information**

| **Supplementary Table 1. Primers used for qRT-PCR** | |  |
| --- | --- | --- |
| **Gene** | **Forward primer** | **Reverse primer** |
| *Hprt1* | GCAAACTTTGCTTTCCCTGGTT | CAAGGGCATATCCAACAACA |
| *Casp3* | GACTGATGAGGAGATGGCTTG | TGCAAAGGGACTGGATGAAC |
| *Casp7* | CCCACTTATCTGTACCGCATG | GGTTTTGGAAGCACTTGAAGAG |
| *Casp8* | AACTTCCTAGACTGCAACCG | TCTCAATTCCAACTCGCTCAC |
| *Gpx4* | GCAATGAGGCAAAACTGACG | CTTGATTACTTCCTGGCTCCTG |
| *Nox4* | TCCAAGCTCATTTCCCACAG | CGGAGTTCCATTACATCAGAGG |
| *Ucp2* | GCATTGGCCTCTACGACTC | AAGCGGACCTTTACCACATC |
| *Gss* | GATCCTGTCCAATAACCCCAG | GCACGCTGGTCAAATATGTTC |
| *Ctsb* | AGACCTGCTTACTTGCTGTG | GGAGGGATGGTGTATGGTAAG |
| *Ncf1* | TCATCCTTCAGACCTATCGGG | ACCTCGCTTTGTCTTCATCTG |
| *Ehd2* | AGCTCAACGACCTAGTGAAAC | TCGCAAAGATGACAGGCAG |
| *Ripk1* | GGAAGGATAATCGTGGAGGC | AAGGAAGCCACACCAAGATC |
| *Ripk3* | TCTTTACTGAGACTCCCGGT | AGTTCCCAATCTGCACTTCAG |
| *Mlkl* | ACTGTGAACTTGGAACCCTG | TGCTGATGTTTCTGTGGAGTG |
| *Tradd* | ACGAACTCACTAGTCTAGCAGAG | AATACCCCAACAGCCACC |
| *Tnf1α* | CTTCTGTCTACTGAACTTCGGG | CAGGCTTGTCACTCGAATTTTG |
| *Tnfrsf1a* | CTCTGCTCTACGAATCACTCTG | CACAGCATACAGAATCGCAAG |
| *Tnfrsf9* | CCTGTGATAACTGTCAGCCTG | TCTTGAACCTGAAATAGCCTGC |
| *Nlrp3* | CTCCAACCATTCTCTGACCAG | ACAGATTGAAGTAAGGCCGG |
| *Il1α* | TGCAGTCCATAACCCATGATC | ACAAACTTCTGCCTGACGAG |
| *Il1β* | ACGGACCCCAAAAGATGAAG | TTCTCCACAGCCACAATGAG |
| *Il6* | CAAAGCCAGAGTCCTTCAGAG | GTCCTTAGCCACTCCTTCTG |
| *Il22* | AGCTTGAGGTGTCCAACTTC | GGTAGCACTGATCTTTAGCACTG |

**Supplementary Figures**

**

**

**Supplementary Figure 1.** Serum T3 levels in mice that have been treated with anti-thyroid drugs. Mice received anti-thyroid treatment *via* drinking water (1% sodium perchlorate monohydrate and 0.05% methomazole), beginning at postnatal day 20 (P20), received a single injection of NaIO_3_ (30 mg/kg, *i.p.*) at P30, and were analyzed for serum T3 levels by ELISA at 3 days post-NaIO_3_ injection. Shown are the serum T3 levels in different groups of mice. Data are presented as mean ± *SEM* for 5-8 mice per group (*** *p* < 0.001).

**
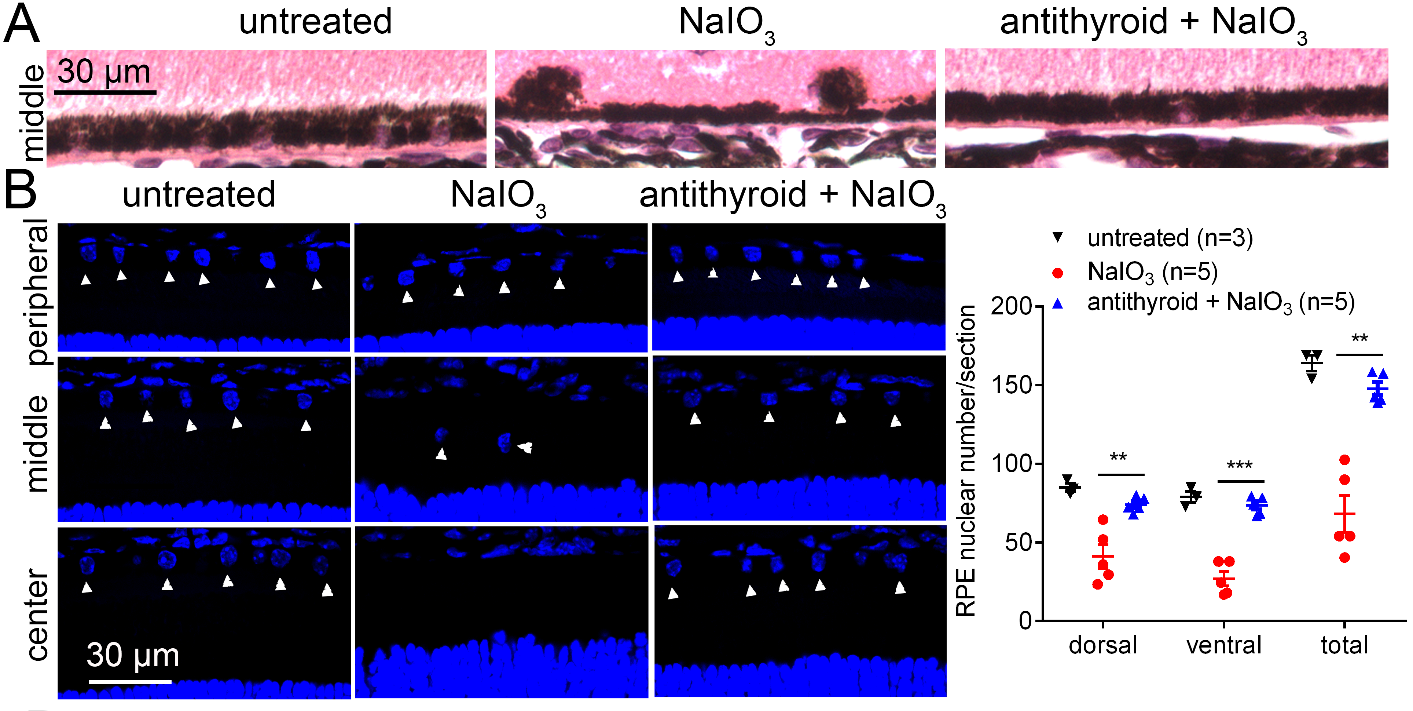
**

**Supplementary Figure 2.** Treatment with anti-thyroid drug protected RPE from cell death induced by NaIO_3_. RPE morphology and cell loss were evaluated on eye cross sections with H&E staining and DAPI labeling at 3 days post-NaIO_3_ injection. **A.** Shown are representative light microscopic images of retinal cross sections with H&E. **B.** Shown are representative images of DABI labeling, and corresponding quantitative analysis of RPE nuclear numbers. Data are presented as mean ± *SEM* for 3-5 mice per group (**, *p* < 0.01, *** *p* < 0.001).

**
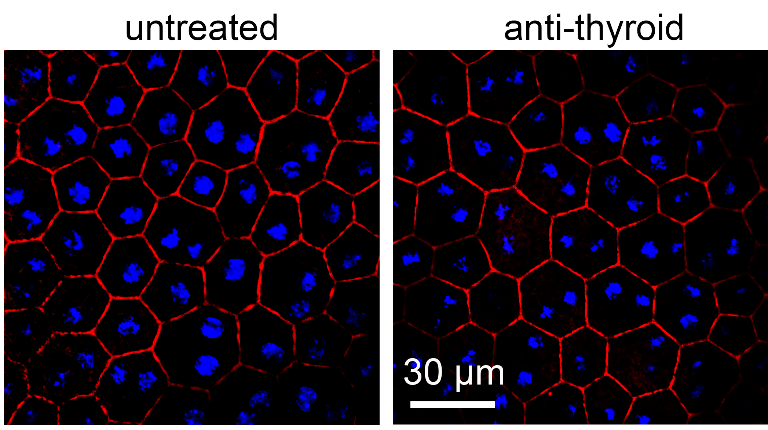
**

**Supplementary Figure 3.** Treatment with anti-thyroid drug alone did not induce significant alterations in RPE morphology in young mice. Mice at P20 received anti-thyroid treatment *via* drinking water (1% sodium perchlorate monohydrate and 0.05% methomazole) for 13 days, and were evaluated for RPE morphology by phalloidin staining and DAPI staining on the RPE whole mounts at the end of the experiments. Shown are representative images of phalloidin labeling and DAPI staining in the treated and untreated mice.


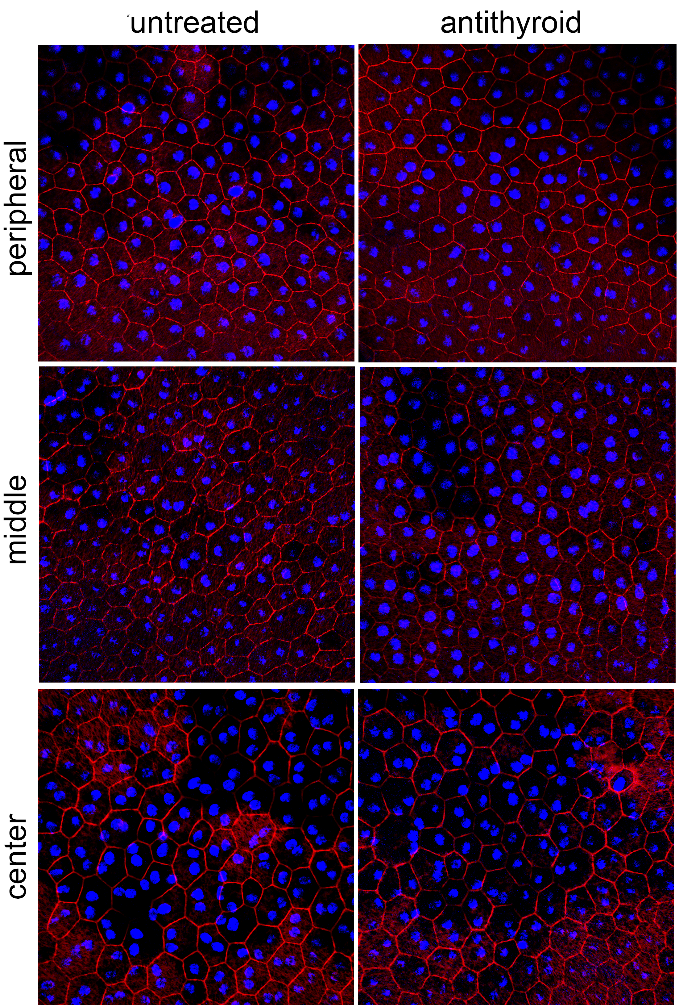


**Supplementary Figure 4.** Treatment with anti-thyroid drug alone did not induce significant alterations in RPE morphology in aged mice. Mice at 17 months received anti-thyroid treatment *via* drinking water (1% sodium perchlorate monohydrate and 0.05% methomazole) for 12 days, and were evaluated for RPE morphology by phalloidin staining and DAPI staining on the RPE whole mounts at the end of the experiments. Shown are representative images of phalloidin labeling and DAPI staining in the treated and untreated mice.
