# Supplementary material for: Inhibition of thyroid hormone signaling protects retinal pigment epithelium and photoreceptors from cell death in a mouse model of age-related macular degeneration
Source: Cell Death Dis. 2020 Jan 13;11(1):24. doi: 10.1038/s41419-019-2216-7 (PMC6957507; doi:10.1038/s41419-019-2216-7)
Supplement: Supplementary file 2 — Supplementary Table 1 [file 41419_2019_2216_MOESM2_ESM.docx]

| **Supplementary Table 1. Primers used for qRT-PCR** | |  |
| --- | --- | --- |
| **Gene** | **Forward primer** | **Reverse primer** |
| *Hprt1* | GCAAACTTTGCTTTCCCTGGTT | CAAGGGCATATCCAACAACA |
| *Casp3* | GACTGATGAGGAGATGGCTTG | TGCAAAGGGACTGGATGAAC |
| *Casp7* | CCCACTTATCTGTACCGCATG | GGTTTTGGAAGCACTTGAAGAG |
| *Casp8* | AACTTCCTAGACTGCAACCG | TCTCAATTCCAACTCGCTCAC |
| *Gpx4* | GCAATGAGGCAAAACTGACG | CTTGATTACTTCCTGGCTCCTG |
| *Nox4* | TCCAAGCTCATTTCCCACAG | CGGAGTTCCATTACATCAGAGG |
| *Ucp2* | GCATTGGCCTCTACGACTC | AAGCGGACCTTTACCACATC |
| *Gss* | GATCCTGTCCAATAACCCCAG | GCACGCTGGTCAAATATGTTC |
| *Ctsb* | AGACCTGCTTACTTGCTGTG | GGAGGGATGGTGTATGGTAAG |
| *Ncf1* | TCATCCTTCAGACCTATCGGG | ACCTCGCTTTGTCTTCATCTG |
| *Ehd2* | AGCTCAACGACCTAGTGAAAC | TCGCAAAGATGACAGGCAG |
| *Ripk1* | GGAAGGATAATCGTGGAGGC | AAGGAAGCCACACCAAGATC |
| *Ripk3* | TCTTTACTGAGACTCCCGGT | AGTTCCCAATCTGCACTTCAG |
| *Mlkl* | ACTGTGAACTTGGAACCCTG | TGCTGATGTTTCTGTGGAGTG |
| *Tradd* | ACGAACTCACTAGTCTAGCAGAG | AATACCCCAACAGCCACC |
| *Tnf1α* | CTTCTGTCTACTGAACTTCGGG | CAGGCTTGTCACTCGAATTTTG |
| *Tnfrsf1a* | CTCTGCTCTACGAATCACTCTG | CACAGCATACAGAATCGCAAG |
| *Tnfrsf9* | CCTGTGATAACTGTCAGCCTG | TCTTGAACCTGAAATAGCCTGC |
| *Nlrp3* | CTCCAACCATTCTCTGACCAG | ACAGATTGAAGTAAGGCCGG |
| *Il1α* | TGCAGTCCATAACCCATGATC | ACAAACTTCTGCCTGACGAG |
| *Il1β* | ACGGACCCCAAAAGATGAAG | TTCTCCACAGCCACAATGAG |
| *Il6* | CAAAGCCAGAGTCCTTCAGAG | GTCCTTAGCCACTCCTTCTG |
| *Il22* | AGCTTGAGGTGTCCAACTTC | GGTAGCACTGATCTTTAGCACTG |
