# Supplementary figures and images for: Inhibition of thyroid hormone signaling protects retinal pigment epithelium and photoreceptors from cell death in a mouse model of age-related macular degeneration
Source: Cell Death Dis. 2020 Jan 13;11(1):24. doi: 10.1038/s41419-019-2216-7 (PMC6957507; doi:10.1038/s41419-019-2216-7)

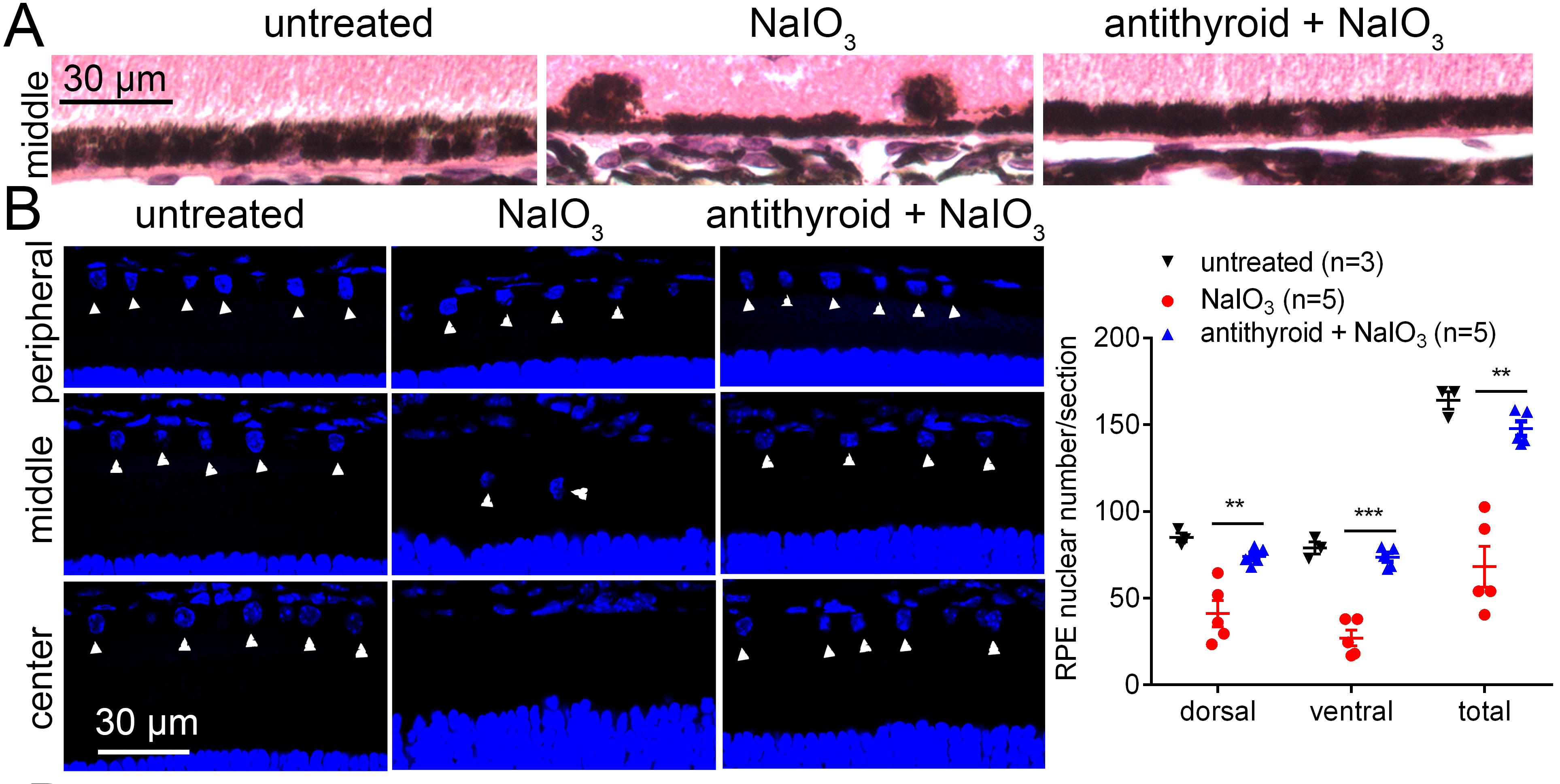

Supplement: Supplementary file 4 — Supplementary Figure 2 [file 41419_2019_2216_MOESM4_ESM.tif]

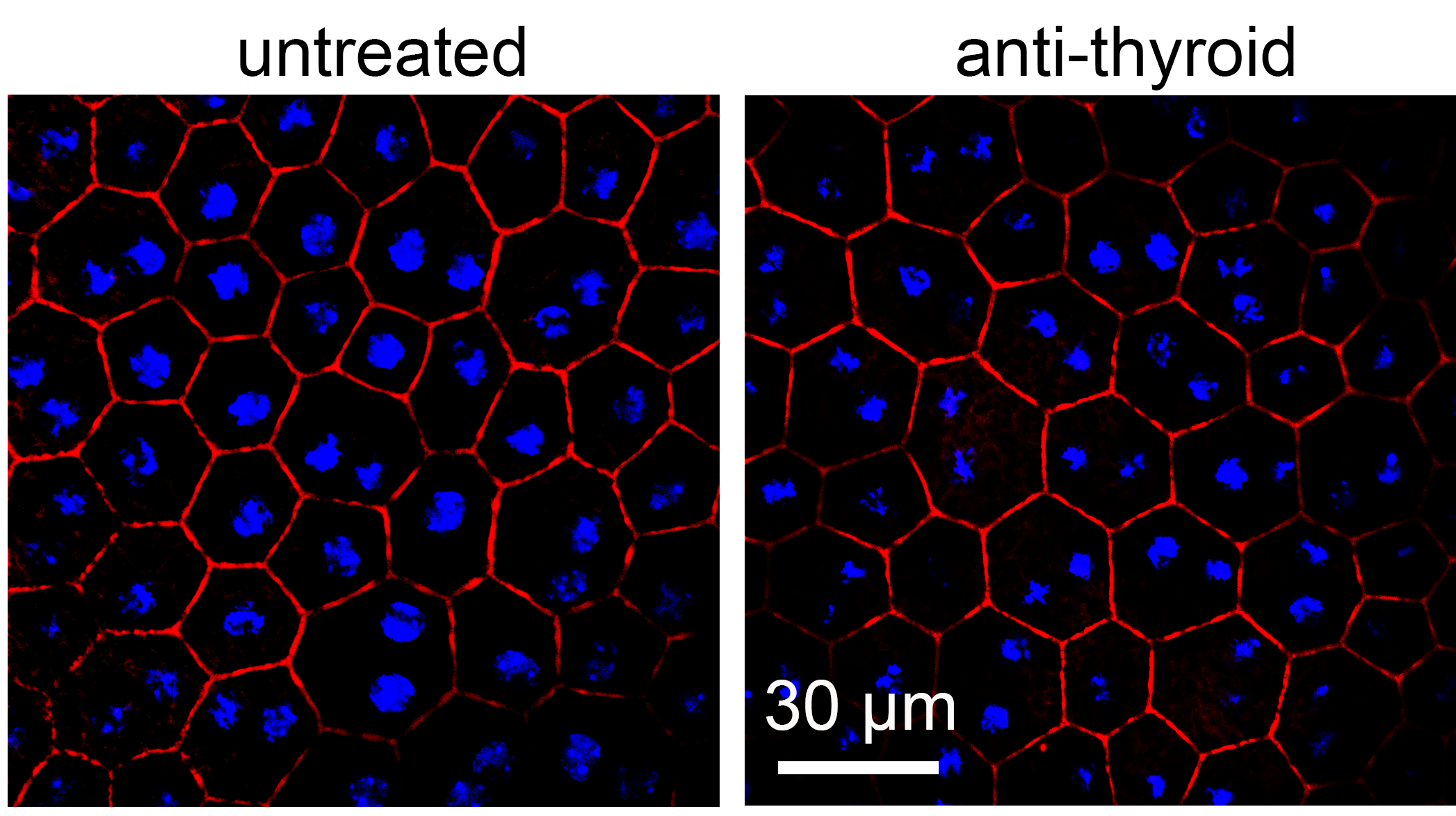

Supplement: Supplementary file 5 — Supplementary Figure 3 [file 41419_2019_2216_MOESM5_ESM.tif]
